# Taurine Attenuates *Streptococcus uberis*-Induced Bovine Mammary Epithelial Cells Inflammation via Phosphoinositides/Ca^2+^ Signaling

**DOI:** 10.3389/fimmu.2019.01825

**Published:** 2019-08-07

**Authors:** Ming Li, Panpan Xi, Yuanyuan Xu, Zhenglei Wang, Xiangan Han, Wenkai Ren, Vanhnaseng Phouthapane, Jinfeng Miao

**Affiliations:** ^1^MOE Joint International Research Laboratory of Animal Health and Food Safety, College of Veterinary Medicine, Nanjing Agricultural University, Nanjing, China; ^2^Chinese Academy of Agricultural Sciences, Shanghai Veterinary Research Institute, Shanghai, China; ^3^Guangdong Provincial Key Laboratory of Animal Nutrition Control, College of Animal Science, Subtropical Institute of Animal Nutrition and Feed, South China Agricultural University, Guangzhou, China; ^4^Biotechnology and Ecology Institute, Ministry of Science and Technology, Vientiane, Laos

**Keywords:** taurine, phosphoinositides, Ca^2+^, *Streptococcus uberis*, inflammation

## Abstract

Taurine may alleviate the inflammatory injury induced by *Streptococcus uberis* (*S. uberis*) infection by regulating intracellular Ca^2+^ levels. However, the underlying mechanisms remain unclear. Infection leads to subversion of phosphoinositides (PIs) which are closely related to Ca^2+^ signaling. In order to investigate whether taurine regulates inflammation by means of PIs/ Ca^2+^ systems, competitive inhibitors of taurine (β-alanine) siTauT, siPAT1, siPLC, siCaN, siPKC, and inhibitors of PLC (U73122), PKC (RO31-8220), and CaN (FK 506) were used. The results indicate that taurine transfers the extracellular nutrient signal for intercellular innate immunity to phosphoinositides without a need to enter the cytoplasm while regulating intracellular Ca^2+^ levels during inflammation. Both the Ca^2+^-PKCα-NF-κB, and Ca^2+^-CaM-CaN-NFAT signaling pathways of *S. uberis* infection and the regulatory roles of taurine follow activation of PIs/Ca^2+^ systems. These data increase our understanding on the mechanisms of multifunctional nutrient, taurine attenuated inflammatory responses caused by *S. uberis* infection, and provide theoretical support for the prevention of this disease.

## Introduction

Bovine mastitis is the most important disease of dairy cattle, leading to enormous production losses (1, 2). *Streptococcus uberis* (*S. uberis)* is an important mastitis causing pathogen due to its ability to adhere to the surfaces of mammary alveoli and internalize into mammary epithelial cells (MECs) thus escaping elimination by the host (3–5). Conventional mastitis control measures including the use of vaccines and antibiotics are ineffective and may lead to resistant superorganisms and drug residue in milk products (6). Regulating the natural defense mechanisms of the mammary gland and/or MECs to reduce the risk or degree of infection may be useful in controlling various udder pathogens and intracellular infections. It has been reported that Panax ginseng extract is able to trigger an adequate immune response demonstrating its protective effect and potential for preventing bovine intramammary infection with *Staphylococcus aureus* (*S. aureus*) (7). Helenalin reduces *S. aureus* intracellular growth and experimental *S. aureus* infection through the inhibition of inflammatory factors secretion (8). Taurine (2-aminoethanesulfonic acid), one of the most abundant free amino acids in most animal tissues has many fundamental biological roles (9). It is suggested that additional administration of taurine and its derivatives may regulate the host's innate immune response and be effective in the treatment or prevention of various topical infections and inflammatory diseases (10–12). Studies in our lab reveal that taurine can increase antioxidant ability, down-regulate inflammatory responses and moderate injury of mammary gland/MECs from *S. uberis* challenge (13–15). The underlying mechanism(s) of how taurine attenuates the inflammatory response of mammary gland challenged by *S. uberis* is confusing and likely complex.

Previous studies established that taurine crosses the cytomembrane via 2 transporters: (1) the high-affinity, low-capacity Na^+^- and Cl^−^-dependent transporter TauT (SLC6A6); (2) the low-affinity, high-capacity H^+^-coupled, pH-dependent, Na^+^-and Cl^−^ -independent transporter PAT1 (SLC36A1) (16, 17). Taurine influx modulates the activity of voltage-dependent Ca^2+^, Na^+^ channels and the activity of the Na^+^/Ca^2+^exchanger which results in the regulation of cytoplasmic free Ca^2+^ concentrations and initiates the triggering of Ca^2+^ signaling (18, 19). Recently, it has been shown that Ca^2+^, as a second messenger, participates in variant bacterial infections (20, 21). Our former study found taurine could attenuate inflammatory injury in mouse MECs after *S. uberis* challenge by regulating intracellular Ca^2+^ levels and the activities of transcription factors NF-κB and NFAT. The exact means by which taurine affects the intracellular Ca^2+^ levels and the relationship between Ca^2+^ changing and inflammatory responses remain unclear.

Phosphoinositides (PIs) form a minor component on the cytosolic side of eukaryotic cell membranes. They mainly consist of phosphatidylinositol (PI) and the 3 polyphosphoinositides, namely phosphatidylinositol 4-phosphate (PI4P), phosphatidylinositol 4,5-bisphosphate [PI(4,5)P2] and phosphatidylinositol 3,4,5-trisphosphate [PI(3,4,5)P3]. The polyphosphoinositides are various phosphorylation states of PI in its headgroup inositol. Research in the last 20 years has disclosed a wide range of biological processes connected with PIs, turning these lipids into one of the most universal signaling entities in cells that play multiple roles in biology (22). Infection leads to the subversion of PIs metabolism (the relative ratios converting among the 4 component of PIs), that facilitate bacterial engulfment and could modulate the inflammatory response (23–25). Turnover among PIs caused by the activation of surface receptors including G-protein-coupled receptors (GPCRs) and receptor tyrosine kinases (RTKs) when cells are exposed to various extracellular stimuli trigger an increase in cytosolic Ca^2+^ concentration. This increase results from Ca^2+^ influx into the cell through Ca^2+^ channels in the plasma membrane and Ca^2+^ efflux from intracellular stores in the organelles (26–28). These phenomena led to our interest in determining if modulation of PIs metabolism plays a key role in taurine attenuation of *S uberis*-induced inflammatory injury in bMECs and if the regulating role of taurine on the inflammatory responses is related to the activition of NF-κB and NFAT mainly by Ca^2+^ mediated signaling pathways in *S. uberis* infection. Herein, we provide insight into the mechanism(s) of taurine attenuation of inflammatory injury in bovine MECs in *S. uberis* infection and the role of phosphoinositides/Ca^2+^ systems in this bioprocess.

## Materials and Methods

### Bacterial Strains

*S. uberis* 0140J, an encapsulated strain, was purchased from ATCC (USA). For experimental use, bacterial strains were streaked onto Todd-Hewitt broth (THB) solid medium containing 2% fetal bovine serum (FBS; Gibco, Gaithersburg, MD, USA) at 37°C for 24 h. A single colony was put into THB fluid medium at 37°C overnight with shaking. Following 10 μL of overnight culture it was transferred to 1 mL fresh THB at 37°C with shaking until the OD_600_ reached 0.5–0.6.

### Cell Culture and Treatment

MAC-T cells were a gift from Dr. Loor (University of Illinois at Urbana-Champaign, Champaign, IL, USA). Culture fluid contained 10% FBS (Gibco, Gaithersburg, MD, USA), 5 mg/L insulin, 1 mg/L hydrocortisone, 5 mg/L transferrin, 5 μM/L ascorbic acid and 5 mM/L sodium acetate (all from Sigma, St. Louis, MO) in a humidified environment with 5% CO_2_:95% air at 37°C.

MAC-T cells were cultured in 6-well plates to 80% confluence, following removal and culture in serum free medium for 4 h. Confluent monolayers were treated with or without 45 mM of taurine (Sigma, St. Louis, MO, USA) for 24 h. For inhibition experiments, 60 mM β-alanine (Sigma, St. Louis, MO, USA), a competitive inhibitor of taurine; 10 μM U73122, an inhibitor of PLC (dissolved in DMF, Selleck, USA); 10 nM RO31-8220, an inhibitor of PKC (dissolved in DMSO, Selleck, USA); and 100 μM FK 506, an inhibitor of CaN (Astellas, Ireland) were used to pre-treat cells for 1 h. The concentration of taurine, FK 506 and β-alanine was based on previous studies and at these concentrations there was no cytotoxicity (13, 29, 30). Dosages of U73122 and RO31-8220 were based on the manufacturer's recommendations. *S. uberis* was added at mid-exponential phase at a multiplicity of infection (MOI) of 10 for 1–4 h at 37°C. Supernatants were collected and cells were trypsinized (Gibco, Gaithersburg, MD, USA) then centrifuged (1,000 rpm for 5 min) resuspended in phosphate-buffered saline (PBS; Hyclone, Logan, UT, USA) or lysed by incubating on ice for 30 min with lysis buffer (Beyotime, Nantong, China). Supernatants were collected by centrifugation at 1,500 rpm for 10 min at 4°C for further analysis.

For RNA interference tests, the MAC-T cells monolayer was transfected with 50 nm specific siTauT, siPAT1, siPLC, siCaN, siPKC (Ribobio, Guangzhou, China) for 72 h using Lipofectamine 3000 (Invitrogen, U.S.A) reagent according to the manufacturer's instructions, and then administrated taurine for another 24 h. Subsequently, cells were infected with *S. uberis* in mid-exponential phase at a MOI of 10 for 3 h at 37°C. Supernatants were collected and the cells were trypsinized (Gibco, Gaithersburg, MD, USA), centrifuged (1,000 rpm for 5 min) and resuspended in phosphate-buffered saline (PBS; Hyclone, Logan, UT, USA) for further analysis.

### Measurement of Intracellular Reactive Oxygen Species (ROS) and Ca^2+^

Intracellular ROS was evaluated by staining MAC-T cells with DCFH-DA, a fluorescent ROS-sensitive indicator that freely permeates cell membranes. Briefly, after incubating with 10 μM DCFH-DA for 30 min at 37°C, cells were washed 3 times in phosphate buffered saline (PBS) and detached. They were collected at 400 × g for 5 min, resuspended in PBS and immediately analyzed by flow cytometry using FACSCanto.

For intracellular Ca^2+^ detection, cells were washed once in HBSS flux buffer (Hank's balanced salt solution) without calcium chloride, chloride, magnesium sulfate, and phenol red. The cells were incubated in 5 μM Fluo-3/AM (Bebytime, Nantong, China) for 30 min at 37°C, washed 3 times with HBSS flux buffer, and immediately analyzed by flow cytometry.

Ten thousand cells per sample were analyzed using CellQuest Pro acquisition and analysis software.

### Western Blotting

Intracellular protein levels were determined by Western blotting analysis. GAPDH (Bioworld, USA) was employed to ensure equal loading. Cells were washed twice in ice-cold PBS, lysed with RIPA buffer (Beyotime, Nantong, China) added PMSF (Beyotime, Nantong, China) by incubating on ice for 30 min in an Eppendorf tube. The supernatants were collected by centrifuging at 12,000 rpm for 10 min at 4°C; protein concentration was determined by bicinchoninic acid assay (BCA) (Bebytime, Nantong, China). The samples were placed in a polyacrylamide gel by electrophoresis and transferred onto PVDF membranes (Millipore, USA). The membranes were blocked with 5% non-fat milk diluted in Tris buffered saline with Tween-20 (TBST) for 2 h at room temperature, and hybridized overnight with primary antibody at 4°C. Primary antibodies were PLCγ1, p-PLCγ1, NCX1, PI3K, p-PI3K, PTEN, p-PTEN, PKCα, p-PKC, IKKα, p-IKKα, IκBα, p-IκBα, GSK-3β, and p-GSK-3β. NCX1 antibodies were purchased from Albanian Broadband Communication (Abcam, Shanghai, China), and others were from Cell Signaling Technology (CST, Massachusetts, USA). Before and after incubation with the secondary antibodies at room temperature for 2 h, the membranes were washed 3 times with TBST. Secondary antibody is horseradish peroxidase (HRP)—conjugated rabbit anti-goat secondary antibody (CST, Massachusetts, USA). The signals were detected by an ECL Western blot analysis system (Tanon, Shanghai, China). Analysis of bands was quantified with Image J software (NIH, USA).

### Enzyme-Linked Immunosorbent Assay (ELISA)

The cell-free supernatant was collected as described above and TNF-α, IL-1β, and IL-6 measured by ELISA kits (Rigor Bioscience, Beijing, China) according to the manufacturer's instructions. For the detection of PIs, the treated cells were collected in PBS with thawing and refreezing four times and centrifuged at 2,000 rpm for 20 min. The concentrations of PIP3, PIP2, IP3, and CaM in supernatant were detected by commercial ELISA kits (Jianglaibio, Shanghai, China) according to the manufacturer's instructions. Briefly, the sample and Streptavidin-HRP was incubated for 60 min at 37°C with gently shanking, and then, washed 3 times followed by addition of solution A and solution B to each well for 10 min at 37°C. Finally, stop solution was added and absorbance detected using a microplate reader (Thermo, USA) at 450 nm.

### Immunofluorescence Staining

Cells were cultured on round coverslips up to 50% confluence. NF-κB-GFP or NFAT-GFP reporter plasmids (Yesen Biology, Shanghai, China) were transfected into cells via Lipofectamine 3000 (Thermo, USA) according to the manufacturer's protocol. After transfection for 6 h, MAC-T cells were pretreated with β-alanine for 1 h and then taurine for another 24 h. Subsequently, *S. uberis* in mid-exponential phase at a MOI of 10 was added. One hour later, the nutrient solution containing Hoechst 33342 (Bebytime, Nantong, China) was used for another 10 min in the dark for nuclear staining. The translocation status of NF-κB or NFAT into the nucleus was detected by confocal microscopy and a fluorescence microscope (Carl Zeiss, LSM 710).

### DNA Binding Activities Analysis of NF-κB and NFAT

The DNA binding activities of transcription factors (NF-κB and NFAT) in each nucleoprotein extraction solution were sensitively quantified based on DNA-Ag nanocluster molecular beacons and exonuclease III-assisted signal amplification strategy. Briefly, 80 nM of probe (NF-κB or NFAT probe) was mixed with different types of nucleoprotein extracting solutions adjusted to 6 μg/mL. Then, protein binding buffer (10 mM PBS, 1 mM Mg(CH_3_COOH)_2_, 10% glycerol, 0.05 mg/mL poly(dI-dC), pH 7.4) was added into the above solution until the volume reached 25 μL. Two microliter of 10 U/μL Exo III and 3 μL of 10 × Exo III reaction buffer were added for digestion for 30 min at 37°C. Fifteen microliter of Ag nanoclusters molecular beacons (AgMBs), 3 μL of DEPC-treated water, and 2 μL of 10 × Exo III reaction buffer was added to the above solution to bring the total volume of the solution to 50 μL. After incubation at 37°C for 30 min, the solution was mixed with 200 μL of 10 mM PBS and fluorescent signals were recorded by Ls55 PerkinElmer fluorescence spectrophotometer (PerkinElmer, America) under excitation at 564 nm. The emission scan range was chosen between 600 and 700 nm. The probe sequences are listed in Table 1.

**Table 1:** DNA sequence.

**Name**	**Primerssequence(5′-3′)**	**Orientation**
**AgMBs**	CCCTTAATCCCCTCGTCAATGCGATCTGATGACG AGGGTGGGGTGGGGTGGGG	
**NF-κB**	GAGGGGACTTTCCAGCCCCACCCCACCCCACC CTCGTCATCAGATACTTA	Anti-sense
**NF-κ B**	ATCTGATGACGAGGGTGGGGTGGGGTGGGGCT GGAAAGTCCCCTC	Sense
**NFAT**	GAGAGAGGAAAATTGCTGATTGCACAGCCCCAC CCCACCCCACCCTCGTCATCAGATACTTA	Anti-sense
**NFAT**	ATCTGATGACGAGGGTGGGGTGGGGTGGGGCT GTGCAATCAGCAATTTTCCTCTCTC	Sense

### Detection of Calcineurin (CaN), *N*-acetyl-β-D-Glucosaminidase (NAGase), Lactic Dehydrogenase (LDH), Inducible Nitric Oxide Synthase (iNOS), Nitric Oxide (NO), and Total Antioxidant Capacity (T-AOC)

The activities or levels of CaN, NAGase, LDH, iNOS and NO were determined using commercial kits purchased from the Nanjing Jiancheng Bioengineering Institute (Nanjing, China), following the manufacturer's instructions. The assay for CaN activity in cells was based on its ability to catalyze disodium 4-nitrophenyl phosphate (PNPP) to generate the chromogenic substrate p-nitrophenol. One unit (U) of CaN activity was defined as the amount that increased absorbance at a wavelength of 405 nm. For the detection of NAGase activity in supernatant, the optical density of paranitrophenol during the reaction at 37°C between 4-methy-lumbelliferyl-N-acetyl-β*-*glucosaminide substrate with the NAGase contained in the analyzed samples was measured at 400 nm. To measure the activity of LDH, the supernatants were mixed with coenzyme 1 in buffer solution for 15 min at 37°C, mixed with 2, 4-dinitrophenylhydrazine. Sodium hydroxide was used to stop the reaction and measured at 450 nm. For the detection of iNOS activity in cells, the sample was incubated with 0.6 mL reaction buffer and combined with an inhibitor of cNOS (6 mmol/L EGTA). This action was terminated after 15 min at 37°C with 10 mmol/L EDTA and 10 mmol/L HEPES buffer. The formation of a colored chemical compound was photometrically measured at 530 nm. NO production was monitored by measuring the nitrite content in culture medium. The color developing agent was mixed with samples at room temperature for 15 min and measured at 550 nm in a microplate reader (Thermo, USA). Sodium nitrite was the standard. The ABTS method was used to measure total antioxidant capacity; the reason is that ABTS is oxidized to ABTS^+^; while the generation of ABTS^+^ is inhibited in the presence of anti-oxidant. The formation of a colored chemical compound was photometrically measured at 520 nm.

### Protein Microarray of Inflammatory Cytokines and Chemokines

Cells supernatants were collected for surveying concentration by BCA utilized for inflammation cytokines/molecules detection by a Quantibody Mouse Inflammation Array Q1 kit (2) (Ray Biotech, Inc, Norcross, GA; Cat. No. QAM-INF- 1) according to the manufacturer's instructions that can semi-quantitatively authenticate 40 factors. The signals, green fluorescence and Cy3 channel, were gathered via a Gene Pix 4000B laser scanner (Bio-Rad, CA) at 555 nm excitation and 565 nm emission and loading using Gene Pix Pro 6.0 microarray analysis software. Semi-quantitative analysis was performed using Ray Biotech mouse Inflammation Array 1 software (QAM-INF-1_Q Analyzer). The mean fluorescence intensities (median values) of the control group were used as standard.

### RNA Extraction and RT-PCR

Total RNA was extracted using TRIZOL reagent (Invitrogen, Carlsbad, CA, USA) and reverse transcribed (RT) into cDNA using PrimeScript^TM^ RT reagent kit (Taraka, Dalian, China). The PCR reaction was in a total volume of 20 μL using a SYBR Premix Ex Taq^TM^ (Taraka, Dalian, China) in which 2 μL cDNA was added as a template. The primer sequences are in Table 2. As an internal control, the same RT products were subjected to PCR in the presence of a second pair of primers specific to GADPH. Analysis of the relative dates of gene expression used the 2^−ΔΔ*Ct*^ method.

**Table 2:** Prime sequence.

**Gene**	**Primerssequence(5′-3′)**	**Orientation**	**Product size(bp)**
**GAPDH**	ATGCTGGTGCTGAGTATGTG	Forward	174
	CAATCTTGAGGGTGTTGTTAT	Reverse	
**TNF-α**	GGGCGGAGTGTAGGAAGTA	Forward	122
	TCATCTGGAGGAAGCGGTA	Reverse	
**IL-1β**	GGCAACCGTACCTGAACCC	Forward	205
	CACGATGACCGACACCACC	Reverse	
**IL-6**	TTCACTCCATTCGCTGTCT	Forward	227
	GTCTCCTTGCTGCTTTCAC	Reverse	

### Statistical Analyses

The dates in this study were computed using statistical software SPSS 19.0 and results were expressed as means ± SEM. Statistical analyses of the date were performed by ANOVA with a *post-hoc* test. *P* < 0.05 was considered statistically significant.

## Result

### Taurine Downregulates the Cytoplasmic Ca^2+^ Concentration in *S. uberis* Challenged MAC-T Cells Through PLCγ1/IP3 Signaling System

**Figure 1:**
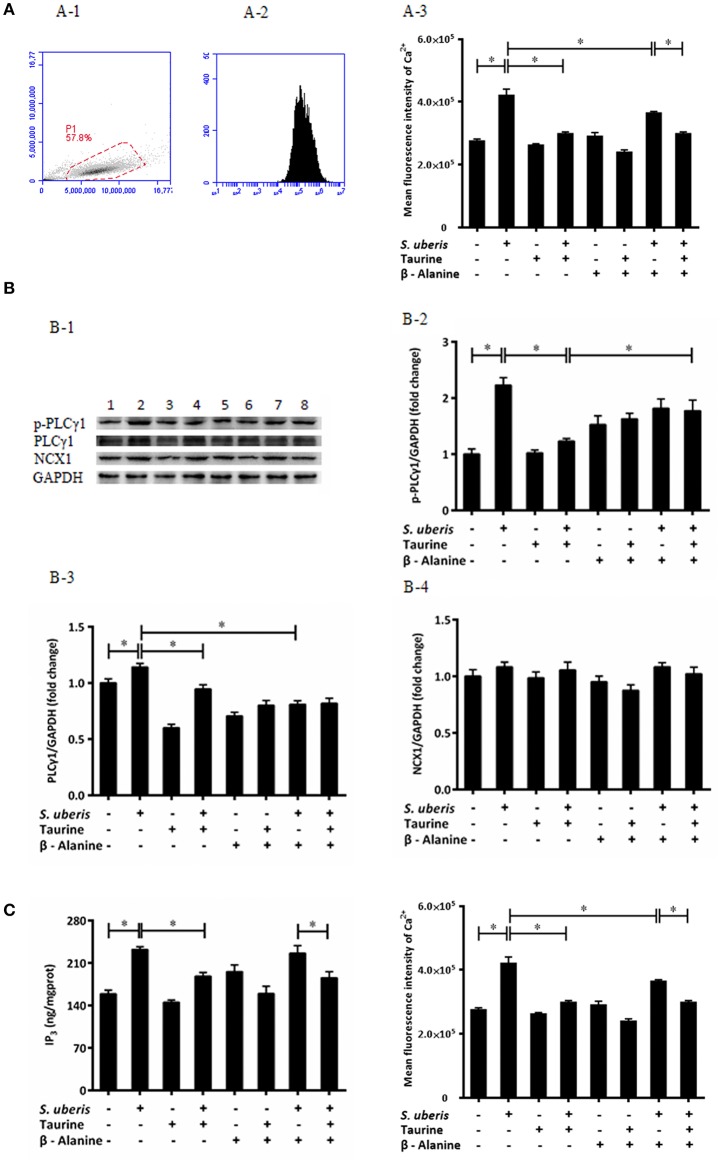
Taurine downregulates cytoplasmic Ca^2+^ concentration in *S. uberis* challenged MAC-T cells through PLCγ1/IP3 signaling system. **(A)** MAC-T cells were pretreated with β-alanine for 1 h and then administrated taurine for 24 h. Subsequently, cells were infected with *S. uberis* in mid-exponential phase at a multiplicity of infection (MOI) of 10 for 3 h at 37°C. Intracellular Ca^2+^ concentration was evaluated by staining cells with Fluo-3 AM. 10,000 cells per sample were analyzed using Cell Quest Pro acquisition and analysis software. Gating of the cell population (A-1), pictorial diagram (A-2), and statistic results (A-3). **(B)** After pretreated with β-alanine and taurine, MAC-T cells were infected with *S. uberis* for 4 h at 37°C. The protein expression of PLCγ1, NCX1, and the phosphorylation levels of PLCγ1 (p-PLCγ1) were determined by Western blot (B-1). Lines 1-8 represent control, *S. uberis*, taurine, *S. uberis* + taurine, β-alanine, β-alanine + taurine, β-alanine + *S. uberis*, β-alanine + taurine + *S. uberis* respectively. To confirm equal protein loading, blots were re-probed with an antibody against GADPH. Results of statistical analysis for p-PLCγ1 (B-2), PLCγ1 (B-3), and NCX1 (B-4) compared to GADPH. **(C)** Two hours after *S. uberis* challenge, the concentration of IP3 was determined using ELISA kits. Statistic results are representative of 3 independent experiments. Data are presented as the means ± SEM. ^*^(*P* < 0.05) = significantly different between the indicated groups.

**Figure 2:**
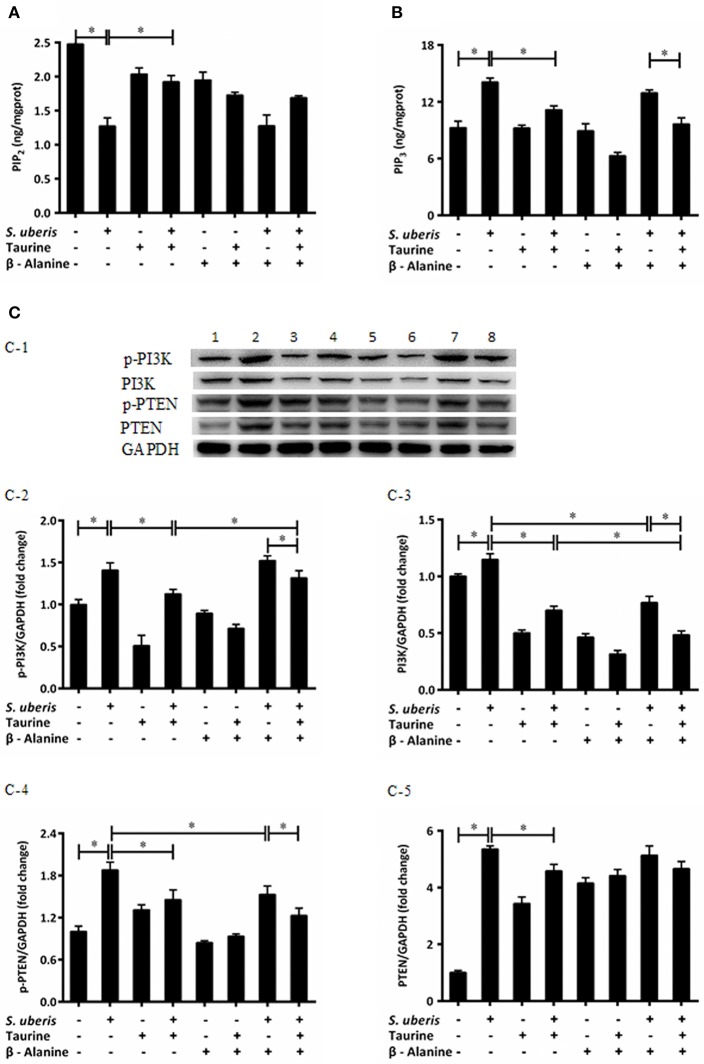
Taurine mediates the intracellular phosphatidylinositols conversion in *S. uberis* challenged MAC-T cells. **(A,B)** MAC-T cells were pretreated with β-alanine for 1 h and then administrated taurine for 24 h. Subsequently, cells were infected with *S. uberis* in mid-exponential phase at a multiplicity of infection (MOI) of 10 for 2 h at 37°C. Cell lysates were used and the concentration of PIP2 **(A)** and PIP3 **(B)** were detected by ELISA kits. **(C)** After pretreated with β-alanine and taurine, MAC-T cells were infected with *S. uberis* for 4 h at 37°C. The protein expression of PI3K (C-3), PTEN (C-5), and their phosphorylation levels p-PI3K (C-2), p-PTEN (C-4) were determined by Western blot (C-1). Lines 1–8 represent control, *S. uberis*, taurine, *S. uberis* + taurine, β-alanine, β-alanine + taurine, β-alanine + *S. uberis*, β-alanine + taurine + *S. uberis*, respectively. To confirm equal protein loading, blots were re-probed with an antibody against GADPH. Results of statistical analysis for p-PI3K (C-2), PI3K (C-3), p-PTEN (C-4), and PTEN (C-5) compared to GADPH. Statistic results are representative of 3 independent experiments. Data are presented as the means ± SEM. ^*^(*P* < 0.05) = significantly different between the indicated groups.

**Figure 3:**
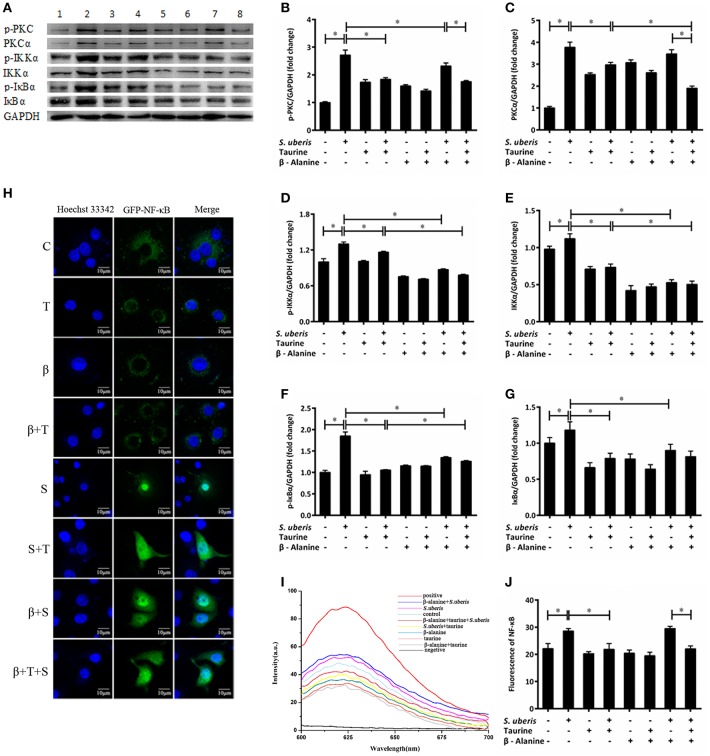
The active PKCα/NF-κB signaling pathway is attenuated by taurine in *S. uberis* challenged MAC-T cells. **(A–G)** MAC-T cells were pretreated with β-alanine for 1 h and then administrated taurine for 24 h. Subsequently, cells were infected with *S. uberis* in mid-exponential phase at a multiplicity of infection (MOI) of 10 for 4 h at 37°C. The protein expression of PKCα **(C)**, IKKα **(E)**, and IκBα **(G)** and their phosphorylation levels p-PKC **(B)**, p-IKKα **(D)**, p-IκBα **(F)** were determined by Western blot **(A)**. Lines 1–8 represent control, *S. uberis*, taurine, *S. uberis* + taurine, β-alanine, β-alanine + taurine, β-alanine + *S. uberis*, β-alanine + taurine + *S. uberis*, respectively. To confirm equal protein loading, blots were re-probed with an antibody against GADPH. Results of statistical analysis for p-PKC **(B)**, PKCα **(C)**, p-IKKα **(D)**, IKKα **(E)**, p-IκBα **(F)**, and IκBα **(G)** compared to GADPH. **(H)** The translocation status of NF-κB into nucleus was detected by confocal. Blue light: nucleus; Green light: NF-κB labeled by GFP; Merge: images that the two lights simultaneously display. C, control; S, *S. uberis*; T,taurine; S + T, *S. uberis* + taurine; β, β-alanine; β + T, β-alanine + taurine; β + S, β-alanine + *S. uberis;* β + T + S, β-alanine + taurine + *S. uberis*. **(I,J)** The contents of NF-κB in nucleoprotein were detected by electrochemistry. The pictorial diagram **(I)** and statistic results **(J)**. Statistic results are representative of three independent experiments. Data are presented as the means ± SEM. ^*^(*P* < 0.05) = significantly different between the indicated groups.

Ca^2+^ is a universal signaling molecule in cells. An increase of the intracellular concentration of Ca^2+^ has been confirmed to be closely related to infection of variant bacteria including *S. uberis*. In the current study, the concentration of intracellular Ca^2+^ was measured by flow cytometry (Figures 1A-1,A-2). As shown in Figure 1A-3, compared with the control, the concentration of intracellular Ca^2+^ in the *S. uberis* challenged group was markedly higher (*P* < 0.05). Taurine administration significantly decreased intracellular Ca^2+^ levels (*P* < 0.05).

In resting cells, the concentration of cytoplasmic Ca^2+^ is normally lower than extracellular and intracellular stores (endoplasmic reticulum, mitochondria). Signaling occurs when the cell is stimulated to release Ca^2+^ from intracellular stores, and/or when calcium enters the cell through plasma membrane ion channels. At the beginning of infection, the sudden increase of cytosolic Ca^2+^ mainly comes from endoplasmic reticulum through the opening of IP_3_-gated Ca^2+^-release channels (31). In order to investigate whether the regulating role of taurine to cytosolic Ca^2+^ concentration connects with IP_3_-gated Ca^2+^-release channels in *S. uberis* challenged bMECs, we determined the IP3 levels in cytoplasm, protein expression of PLCγ1 and its phosphorylation levels (Figure 1B-1) as this enzyme catalyzes the hydrolysis of PIP2 to generate the second messenger IP3. A considerably up-regulated PLCγ1 and its phosphorylation levels were observed between the control and *S. uberis* infection groups (*P* < 0.05). Pre-treatment with taurine significantly reduced this change (*P* < 0.05) (Figures 1B-2, **3**). ELISA results also showed that cytosolic IP3 concentration increased after *S. uberis* challenge. Taurine pre-treatment subverted this change (Figure 1C).

**Figure 4:**
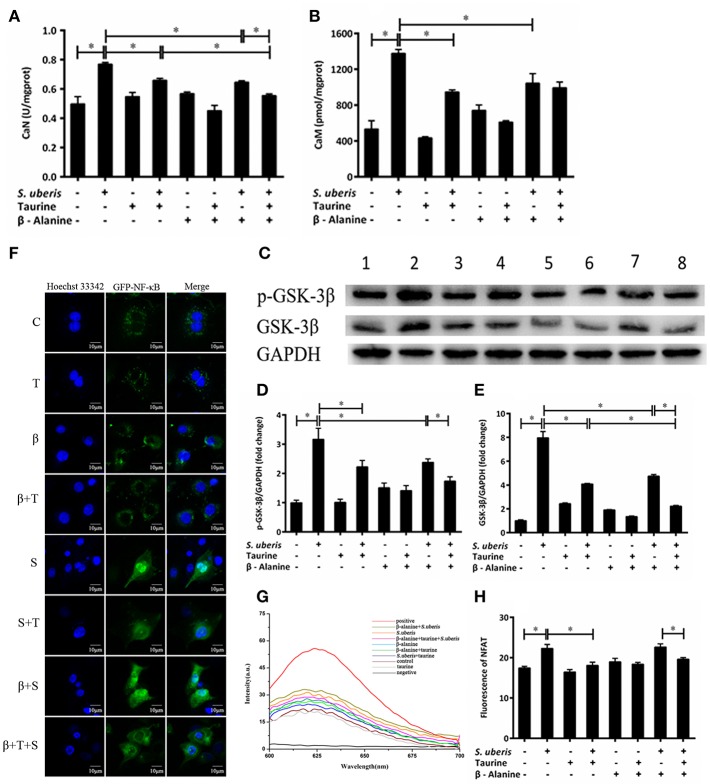
The active CaN/NFAT signaling pathway is attenuated by taurine in *S. uberis* challenged MAC-T cells. **(A–G)** MAC-T cells were pretreated with β-alanine for 1 h and then administrated taurine for 24 h. Subsequently, cells were infected with *S. uberis* in mid-exponential phase at a multiplicity of infection (MOI) of 10 for 4 h at 37°C. **(A,B)** The activities of CaN and the concentration of CaM were determined by commercial kits. **(C)** The protein expression of GSK-3β and its phosphorylation level p-GSK-3β were determined by Western blot. Lines 1–8 represent control, *S. uberis*, taurine, *S. uberis* + taurine, β-alanine, β-alanine + taurine, β-alanine + *S. uberis*, β-alanine + taurine + *S. uberis*, respectively. To confirm equal protein loading, blots were re-probed with an antibody against GADPH. Results of statistical analysis for p-GSK-3β **(D)** and GSK-3β **(E)** compared to GADPH. **(F)** The translocation status of NFAT into nucleus was detected by confocal microscopy. Blue light: for the nucleus; Green light: for the NFAT labeled by GFP; Merge: images that the two lights simultaneously display. C, control; S, *S. uberis*; T, taurine; S + T, *S. uberis* + taurine; β, β-alanine; β + T, β-alanine + taurine; β + S, β-alanine + *S. uberis;* β + T + S, β-alanine + taurine + *S. uberis*. **(G,H)** The contents of NFAT in nucleoprotein were detected by electrochemistry. The pictorial diagram **(G)** and statistic results **(H)**. Statistic results are representative of three independent experiments. Data are presented as the means ± SEM. ^*^(*P* < 0.05) = significantly different between the indicated groups.

Na^+^/Ca^2+^ exchanger (NCX) is a class of bidirectional ion transporter that couples the translocation of Ca^2+^ in one direction with that of Na^+^ in the opposite direction and thus plays an important role in the regulation of intracellular Ca^2+^. Herein, we used Western blot to assay the expression of NCX1, one of the NCX isoforms, and found there were no significantly differences among different groups (Figure 1B-4). This data is consistent with a previous study that found that taurine mainly inhibits the influx of Ca^2+^ through NCX and has no effect on the rate of Ca^2+^ efflux from cytoplasm (32).

The inhibitory role of taurine on increasing intracellular Ca^2+^ is very complex. It is clear that taurine can modulate the activity of various ion channels to regulate Ca^2+^ homeostasis. These factors affect taurine transport and/or directly bind with different channel proteins and membrane lipids (33, 34). To evaluate if the translocation of taurine is necessary and these ions link with the transmembrane movement of Ca^2+^, its analog β-alanine was used to compete with it. The results suggest that β-alanine administration did not significantly influence the down-regulatory role of taurine to cytosolic Ca^2+^ levels, PLCγ1 protein expression, and IP3 concentration. There were no significant differences between the taurine + *S. uberis* and β-alanine + taurine + *S. uberis* groups (*P* > 0.05) (Figures 1A-3,B-3,B-4,C). These data indicate that taurine could significantly decrease intracellular Ca^2+^ levels through the PLCγ1/IP3 signaling system after *S. uberis* challenge and taurine may be not enter the cytoplasm in this bioprocess.

### Taurine Mediates Intracellular Phosphatidylinositol Conversion in *S. uberis* Challenged MAC-T Cells

IP3 can be hydrolyzed by PLC from PIP2 post infection. Additionally, there is interconversion among different PIs when cells are attacked. The conversion of PIP2 and PIP3 act on internal membranes to control and initiate complex intracellular signaling transduction pathways (22). To determine whether taurine reduced IP3 in *S. uberis* infection linked with conversion of PIP2 and PIP3, PIP2, and PIP3 were evaluated by ELISA kits. Enzyme expression and phosphorylation levels of PI3K, PTEN, which catalyze the exchange of PIP2 and PIP3, were measured by Western blot. The results showed that PIP3 was significantly increased in *S. uberis* challenged cells. Taurine weakens this increase compared with controls. To the contrary, PIP2 was markedly decreased in MAC-T cells in *S. uberis* infection. Taurine pre-treatment increased its values (*P* < 0.05). Higher PIP2 and lower PIP3 concentrations were present in taurine + *S. uberis* groups than in *S. uberis* challenge only groups (*P* < 0.05). There were no significantly differences between the taurine + *S. uberis* and β-alanine + taurine + *S. uberis* groups (*P* > 0.05) (Figures 2A,B).

**Figure 5:**
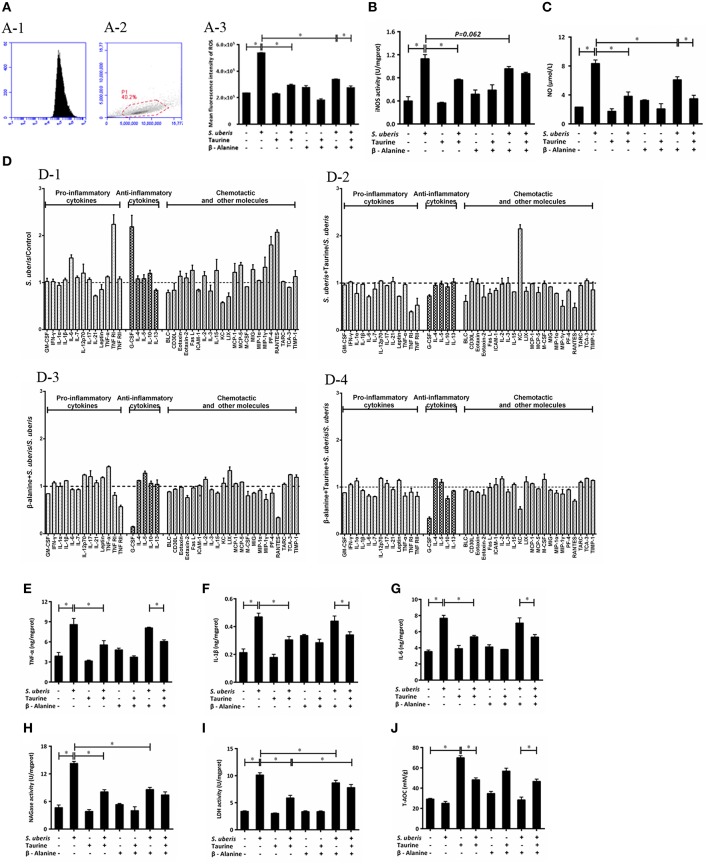
Effect of taurine on inflammatory response in *S. uberis* challenged MAC-T cells. MAC-T cells were pretreated with β-alanine for 1 h and then administrated taurine for 24 h. Subsequently, cells were infected with *S. uberis* in mid-exponential phase at a multiplicity of infection (MOI) of 10 for 4 h at 37°C. **(A)** Intracellular ROS content was evaluated by staining cells with DCFH-DA. 10,000 cells per sample were analyzed using CellQuest Pro acquisition and analysis software. Gating of the cell population (A-1), pictorial diagram (A-2) and statistic results (A-3). **(B,C)** The activities or levels of iNOS and NO in cells or supernatant were determined using commercial kits. **(B)**: iNOS activity, **(C)**: NO level. Statistic results are representative of three independent experiments. Data are presented as the means ± SEM. ^*^(*P* < 0.05) = significantly different between the indicated groups. **(D)** Cell culture supernatants were collected and 300 μg of protein of each sample were used for cytokine expression using a Quantibody Mouse Inflammation Array 1 kit. The array was designed to quantitatively detect 40 cytokines (pro-inflammatory cytokines, anti-inflammatory cytokines, chemotactic and other molecules) simultaneously. The mean fluorescence intensities (median values) of control groups or treated groups were chosen as standards. For (D-1): *S. uberis* vs. Control; (D-2): *S. uberis* + Taurine vs. *S. uberis*; (D-3): β-alanine + *S. uberis* vs. *S. uberis* and (D-4): β-alanine + Taurine + *S. uberis* vs. *S. uberis*. Statistic results are representative of three independent experiments. **(E–G)** The production of cytokines in the supernatant was measured by ELISA commercial kits. For **(E)**: TNF-α; **(F)**: IL-1β and **(G)**: IL-6. **(H,I)** The activities NAGase and LDH in supernatant were determined using commercial kits. **(H)**: NAGase activity and **(I)**: LDH activity. **(J)** The cells were used to detect the ability of antioxidant capacity (T-AOC) by a commercial kit. Statistic results are representative of 3 independent experiments. Data are presented as the means ± SEM. ^*^(*P* < 0.05) = significantly different between the indicated groups.

Phosphatidylinositol 3-kinases (PI3Ks) are key molecules that participate in PIs cytoplasmic metabolism and regulate several key events in the inflammatory response. They selectively phosphorylate PIP2 to PIP3 in the 3-position of the inositol ring. On the contrary, PIP3 is dephosphorylated at the 3-position to re-form PIP2 by the enzyme PTEN (22, 25). So protein expressions and phosphorylation levels of PI3K and PTEN were determined (Figures 2C-1). Exposure of MAC-T cells to *S. uberis* significantly (*P* < 0.05) increases PI3K and PTEN expression and their phosphorylation levels. Taurine reduces the role of *S. uberis* infection (*P* < 0.05). β-alanine has no effect on the decreased PTEN and phosphorylation of PI3K and PTEN (*P* < 0.05) (Figures 2C-2,C-4,C-5), but further decreases the expression of PI3K caused by *S. uberis* infection (*P* > 0.05) (Figure 2C-3). Together, these results suggest that taurine may influence intracellular phosphatidylinositol conversion. Analog β-alanine has almost no effect on this role of taurine in *S. uberis* challenged MAC-T cells.

### The Active PKCα/NF-κB Signaling Pathway Is Attenuated by Taurine in *S. uberis* Challenged MAC-T Cells

Ca^2+^ is linked to the inflammatory response mainly through 2 signaling pathways. One is the activation of PKC by a sudden increase in endoplasmic Ca^2+^ with subsequent activation of nuclear factor-κB (NF-κB); the other is the activation of nuclear factor in activated T cells (NFAT) by CaN (35, 36). Our previous studies suggest that both NF-κB and NFAT may be involved in *S. uberis* induced inflammation in mouse MECs and taurine has a positive regulatory role on the inflammatory response (14). In *S. uberis* infection of bovine MECs, following Ca^2+^ release to cytoplasm PKCα/NF-κB and CaN/NFAT, signaling pathways are activated and induce an intensive inflammatory response. Taurine regulates this process by modulating these changes. Key adaptors, nuclear factors NF-κB and NFAT, inflammatory factors/mediators, and markers to cell injury are assessed in detail in this study. As shown in Figure 3, the expression of PKC, IKKα, and IκBα and their phosphorylation levels were considerably up-regulated in MAC-T cells in response to *S. uberis* and these changes decrease in response to taurine (Figures 3B–G) (*P* < 0.05). β-alanine had a similar effect on IKKα and IκBα protein expression and phosphorylation (Figures 3D–G) (*P* < 0.05). The production p-PKC is also reduced by pre-treatment with β-alanine (Figure 3B) (*P* < 0.05). Co-administration of taurine and β-alanine further lower PKCα and IKKα expression and the phosphorylation of IKKα, but increase IκBα when compared with the taurine + *S. uberis* groups (Figures 3C–F) (*P* < 0.05).

NF-κB controls many genes involved in inflammation and sequesters cytoplasmic IκB. After IκB phosphorylation and detachment, free NF-κB dimers may enter the nucleus. This process can be quantified by cytochemical methods. In the current study, confocal observation demonstrated green fluorescence in the nucleus of control cells. Almost all of this fluorescence appeared in the nucleus 1 h after *S. uberis* challenge. Taurine administration attenuated the translocation of NF-κB. In the taurine + *S. uberis* group, the green fluorescence was present both in the nucleus and cytoplasm (Figure 3H). To quantify the translocation levels of NF-κB, we extracted nuclear protein using a strategy based on DNA-Ag nanocluster molecular beacons and an exonuclease III-assisted signal. As shown in Figure 3J, infection with *S. uberis* enhanced the translocation level of NF-κB to nucleus and taurine reversed this change. These data established that the PKCα/NF-κB signaling pathway is activated by *S. uberis* and the attenuation of this activity may be one of the mechanisms of taurine-associated anti-inflammation in *S. uberis* induced mastitis.

### The Active CaN/NFAT Signaling Pathway Is Attenuated by Taurine in *S. uberis* Challenged MAC-T Cells

CaN is a Ca^2+^/CaM dependent phosphatase that is particularly relevant in the modulation of nuclear signaling events, proceeding through the family of NFAT transcription factors. Its activity is markedly increased in response to *S. uberis*, whereas the addition of taurine inhibits this increase (*P* < 0.05). β-alanine significantly inhibits the activity of CaN in response to *S. uberis* challenge. Co-administration of taurine further lowers the activation of CaN (*P* < 0.05) (Figure 4A). CaM increases significantly after *S. uberis* infection (*P* < 0.05). Taurine, β-alanine, individually or together, greatly reduce its expression (*P* < 0.05). There was no significant difference between the taurine + *S. uberis* and β-alanine + taurine + *S. uberis* groups (*P* > 0.05) (Figure 4B). Glycogen synthase kinase 3 (GSK-3) regulates the NFAT phosphorylation state and export from the nucleus (37). We quantified GSK-3β and its phosphorylation by Western blot and found they were increased in response to *S. uberis* (*P* < 0.05) and taurine decreased them (Figures 4C–E). Confocal microscopy results showed that, similar to NF-κB, more green fluorescence appeared in the nuclear region in infected cells than in controls. Taurine pre-treatment reduces the translocation of NFAT (Figure 4F). This was confirmed by quantitative detection (Figure 4H). The results suggested that the active CaN/NFAT signaling pathway can be attenuated by taurine in *S. uberis* challenged MAC-T cells.

### Taurine Inhibits the Inflammatoty Response and Cell Injury in *S. uberis* Challenged MAC-T Cells

Inflammatory mediators and cell injury indexes were measured. Flow cytometry detection via staining cells with 10 μM DCFH-DA indicate that intracellular ROS level increased after *S. uberis* co-culture with MAC-T cells (Figures 5A-1,A-2); significant differences were observed when bacterial challenged groups were compared to controls (*P* < 0.05). Pre-treatment with taurine or/and β-alanine decreased its production when compared with the *S. uberis* challenged group (*P* < 0.05). No significant difference was seen between the taurine + *S. uberis* and β-alanine + taurine + *S. uberis* groups (*P* > 0.05) (Figure 5A-3).

Intracellular iNOS activities and NO were detected by commercial kits. *S. uberis* infection significantly elevated iNOS activity; NO and taurine significantly reduced these increases (*P* < 0.05). β-alanine also lessened NO dramatically and iNOS activity to a lesser extent. Co-administration of β-alanine and taurine had no additive effect when compared with the taurine + *S. uberis* group (*P* > 0.05) (Figures 5B,C).

To determine the influence of taurine on the production of inflammatory cytokines/molecules, the Quantibody Mouse Inflammation Array Q1 kit was used to semi-quantitatively assay 40 cytokines/molecules. These molecules are divided into 3 groups: pro-inflammatory cytokines (13), anti-inflammatory cytokines (5) and chemotactic and other molecules in culture supernatants (22) (Figure 5D). Data indicate the secretion of the majority of these molecules (28/40) are increased (fold change > 1) 4 h after *S. uberis* challenge; for 12/40 molecules the fold change (>1.2) is significant. Of the 28 changed molecules, 10/13 are pro-inflammatory cytokines, 4/5 are anti-inflammatory cytokines and the rest are chemotactic and other molecules (Figure 5D-1). Taurine pre-treatment of the *S. uberis* infected cells results in decreased secretion of 33/40 molecules (fold change < 1), including 10/13 pro-inflammatory cytokines, 4/5 anti-inflammatory cytokines and 19/22 chemotactic and other molecules; 13 of these 33 molecules had a significant reduction (fold change < 0.8) (Figure 5D-2). In Figure 5D-3, β-alanine pre-treatment of *S. uberis* infected cells results in the decreased secretion of 20/40 molecules (fold change < 1), including 6/13 pro-inflammatory cytokines, 1/5 anti-inflammatory cytokines, and 13/22 chemotactic and other molecules. Only five had a significant reduction (fold change < 0.8). Twenty four molecules decreased (fold change < 1), including 8/13 pro-inflammatory cytokines, 3/5 anti-inflammatory cytokines and 13/22 chemotactic and other molecules (Figure 5D-4, where 5 had a significant reduction (fold change < 0.8). These results suggest that taurine may reduce the secretion of inflammation-associated factors induced by *S. uberis* in MAC-T cells while the effects of β-alanine are not so obvious compared with taurine.

In our previous studies, we focused on TNF-α, IL-1β, and IL-6, 3 pro-inflammatory cytokines that are expressed at elevated levels following activation of nuclear transcription factors and are closely related to the inflammatory response. In the current study, ELISA testing determined that TNF-α, IL-1β, and IL-6 expression were all considerably up-regulated in MAC-T cells in response to *S. uberis* infection; the opposite results were observed in taurine pre-treatment groups compared with the *S. uberis* group (*P* < 0.05) Figures 5E–G.

The cell injury biomarkers NAGase and LDH in the cell culture supernatants were significantly higher in *S. uberis* infection groups. Pre-treatment with taurine or/and β-alanine down-regulated their activities (*P* < 0.05). There were no significant differences between the taurine + *S. uberis* and β-alanine + taurine + *S. uberis* groups for NAGase (*P* > 0.05) Figures 5H,I. β-alanine also did not inhibit taurine induced T-AOC increasing in *S. uberis* challenged cells (Figure 5J). These data indicate that taurine inhibits inflammation and cell injury in *S. uberis* challenged MAC-T cells.

### The Influence of TauT and PAT1 Expression on the Effect of Taurine on *S. uberis* Induced Inflammation in MAC-T Cells

**Figure 6:**
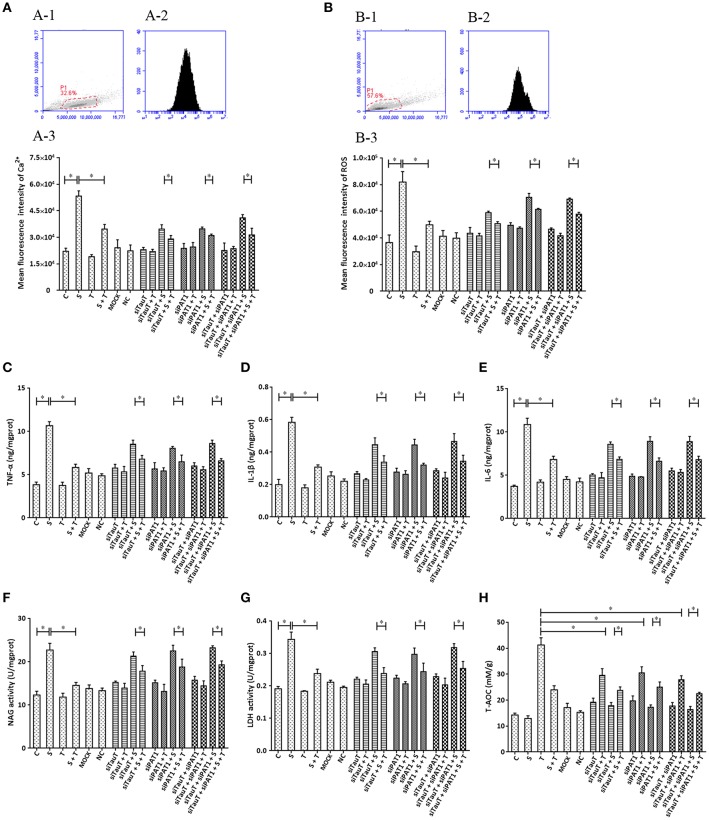
The influence of interference in the expression of TauT expression and PAT1 on the effect of taurine on inflammatory responses induced by *S. uberis* in MAC-T cells. MAC-T cells were transfected with the siTauT or/and siPAT1 for 72 h and then administrated taurine for 24 h. Subsequently, cells were infected with *S. uberis* in mid-exponential phase at a multiplicity of infection (MOI) of 10 for 3 h at 37°C. Intracellular Ca^2+^ concentration was evaluated by staining cells with Fluo-3 AM. 10,000 cells per sample were analyzed using Cell Quest Pro acquisition and analysis software. Gating of the cell population (A-1), pictorial diagram (A-2), and statistic results (A-3). (B-H) Cells were infected with *S. uberis* in mid-exponential phase at a multiplicity of infection (MOI) of 10 for 4 h at 37°C. **(B)** Intracellular ROS content was evaluated by staining cells with DCFH-DA. 10,000 cells per sample were analyzed using CellQuest Pro acquisition and analysis software. Gating of the cell population (B-1), pictorial diagram (B-2), and statistic results (B-3). The production of cytokines in the supernatant was measured by ELISA kits. For **(C)**: TNF-α; **(D)**: IL-1β and **(E)**: IL-6. The activities NAGase and LDH in supernatant were determined using commercial kits. For **(F)**: NAGase activity and **(G)**: LDH activity. **(H)** The cells were used to detect the ability of antioxidant capacity (T-AOC) by a commercial kit. Statistic results are representative of three independent experiments. Data are presented as the means ± SEM. ^*^(*P* < 0.05) = significantly different between the indicated groups.

Taurine uptake across the cell membrane is via two transporters: H^+^-coupled PAT1 (SLC36A1) and Na^+^- and Cl^−^-dependent TauT (SLC6A6). We explored the possibility that taurine regulation of *S. uberis* challenged cells might not depend on entry into the cytoplasm. Cells were transfected with siTauT or/and siPAT1 to inhibit the expression of these two transporters. The results showed that cells co-cultured with siTauT or/and siPAT1 and then administrated taurine had significantly decreased intracellular Ca^2+^ concentrations after *S. uberis* challenge. The inhibition rates for siTauT, siPAT1, and siTauT + siPAT1 were 16.06, 10.85, and 23.59%, respectively (*P* < 0.05), lower then when taurine is directly added (34.97% (*P* < 0.05) Figure 6A. The inhibition rate of ROS due to taurine in *S. uberis* infection is 39.18% (*P* < 0.05). With siTauT, siPAT1, or siTauT + siPAT1, the percent inhibition is 13.96, 12.74, and 16.38%, respectively (*P* < 0.05) Figure 6B. In association with TNF-α, IL-1β, and IL-6, taurine reduced secretion after *S. uberis* challenge; the suppression ratio is 45.38, 47.30, and 37.17%, respectively (*P* < 0.05). The ratio is higher than that of cells pretreated with siTauT, siPAT1, or siTauT + siPAT1 (Figures 6C–E). Similar changes were observed regarding the activities of NAGase and LDH in supernatant (Figures 6F,G). The total intracellular antioxidant capacity (T-AOC) was determined by a commercial kit. The data established that siTauT, siPAT1, or siTauT + siPAT1 may significantly decrease the content of T-AOC in cells with taurine (*P* < 0.05) (Figure 6H). These data suggest that downregulated TauT and PAT1 expression reduce taurine uptake, although its inhibition related to *S. uberis* inflammation persists.

### The Influence of Expression of PLC, CaN, and PKC on the Effect of Taurine on Inflammatory Responses Induced by *S. uberis* in MAC-T Cells

**Figure 7:**
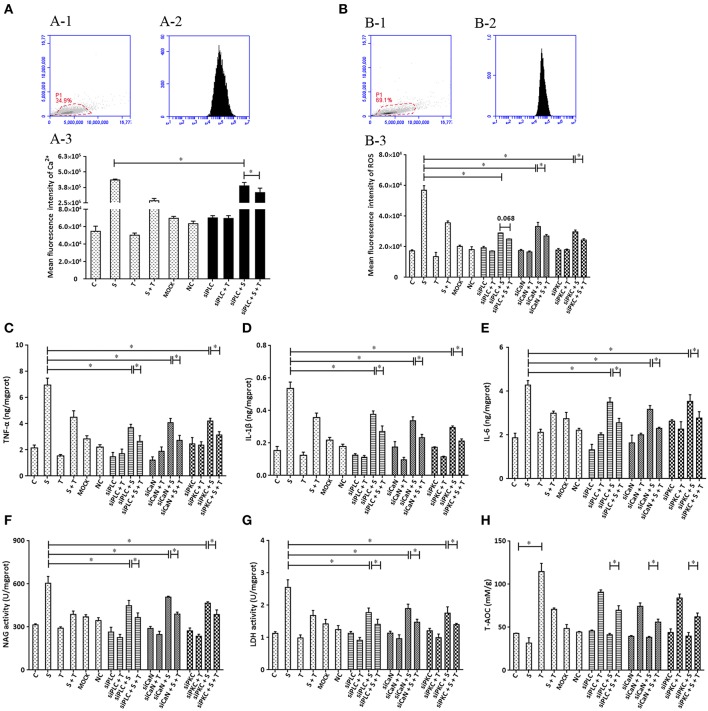
The influence of interference in the expression of PLC, CaN, and PKC on the effect of taurine on inflammatory responses induced by *S. uberis* in MAC-T cells. MAC-T cells were transfected with the siPLC, siCaN, or siPKC for 72 h and then administrated taurine for 24 h. Subsequently, cells were infected with *S. uberis* in mid-exponential phase at a multiplicity of infection (MOI) of 10 for 3 h at 37°C. Intracellular Ca^2+^ was evaluated by staining cells with Fluo-3 AM. 10,000 cells per sample were analyzed using Cell Quest Pro acquisition and analysis software. Gating of the cell population (A-1), pictorial diagram (A-2), and statistic results (A-3). **(B–H)** Cells were infected with *S. uberis* in mid-exponential phase at a multiplicity of infection (MOI) of 10 for 4 h at 37°C. **(B)** Intracellular ROS content was evaluated by staining cells with DCFH-DA. 10,000 cells per sample were analyzed using CellQuest Pro acquisition and analysis software. Gating of the cell population (B-1), pictorial diagram (B-2), and statistic results (B-3). The production of cytokines in the supernatant was measured by ELISA kits. For **(C)**: TNF-α; **(D)**: IL-1β and **(E)**: IL-6. The activities NAGase and LDH in supernatant were determined using commercial kits. **(F)**: NAGase activity and **(G)**: LDH activity. **(H)** The cells were used to detect the ability of antioxidant capacity (T-AOC) by a commercial kit. Statistic results are representative of 3 independent experiments. Data are presented as the means ± SEM. ^*^(*P* < 0.05) = significantly different between the indicated groups.

To further confirm that phosphoinositides/Ca^2+^ systems mediate inflammatory signals participate in the inhibition of taurine on *S. uberis* induced inflammation, special interfering RNA targeting to PLC (isoform PLCγ1) was used. As shown in Figure 7, siPLC reduces the increase of intracellular Ca^2+^ in the face of *S. uberis* infection (*P* < 0.05). Taurine also decreases the sharp elevation resulting from *S. uberis* challenge regardless of the presence or absence of siPLC (*P* < 0.05). Inhibition by siPLC was 13.92 and 38.43% (Figure 7A-3). SiPLC downregulates *S. uberis* induced intracellular ROS; TNF-α, IL-1β, and IL-6; NAGase, LDH. T-AOC is upregulated (Figures 7B–H). Pretreatment with siPLC significantly decreases the suppressive role of taurine on the inflammatory response and cell injury indexes. When comparing *S. uberis* challenge only groups with taurine + *S. uberis* groups, the suppression ratio is 37.37% for ROS (Figure 7B); 35.42% for TNF-α (Figure 7C), 33.53% for IL-1β (Figure 7D), 30.03% for IL-6 (Figure 7E); 35.92% for NAGase (Figure 7F), and 34.19% for LDH (Figure 7G). These were higher than those between the siPLC + *S. uberis* and siPLC + taurine + *S. uberis* groups. The suppression ratio was 13.49% for ROS (Figure 7B); 28.53% for TNF-α (Figure 7C), 28.10% for IL-1β (Figure 7D), 26.97% for IL-6 (Figure 7E); 18.43% for NAGase (Figure 7F), and 20.54% for LDH (Figure 7G). T-AOC was markedly improved by taurine administration (*P* < 0.05). *S. uberis* challenge attenuated these changes. Pretreatment of cells with siPLC followed by *S. uberis challenge* increased T-AOC levels compared to the *S. uberis* challenge only group (31.66 ± 6.19 vs. 41.21 ± 1.63) (*P* > 0.05) (Figure 7H). Taken together, these data provide evidence that PLC/phosphoinositides/Ca^2+^ mediated signaling pathways may participate in the anti-inflammatory role of taurine. Two key downstream adaptors, CaN and PKC, may initiate the activation of NFAT and NF-κB as target proteins for siRNA (Figures 7B–H).

### The Influence of the Inhibitors of PLC (U73122), CaN (FK506), and PKC (RO31-8220) on the Effect of Taurine on Inflammatory Responses Induced by *S. uberis* in MAC-T Cells

**Figure 8:**
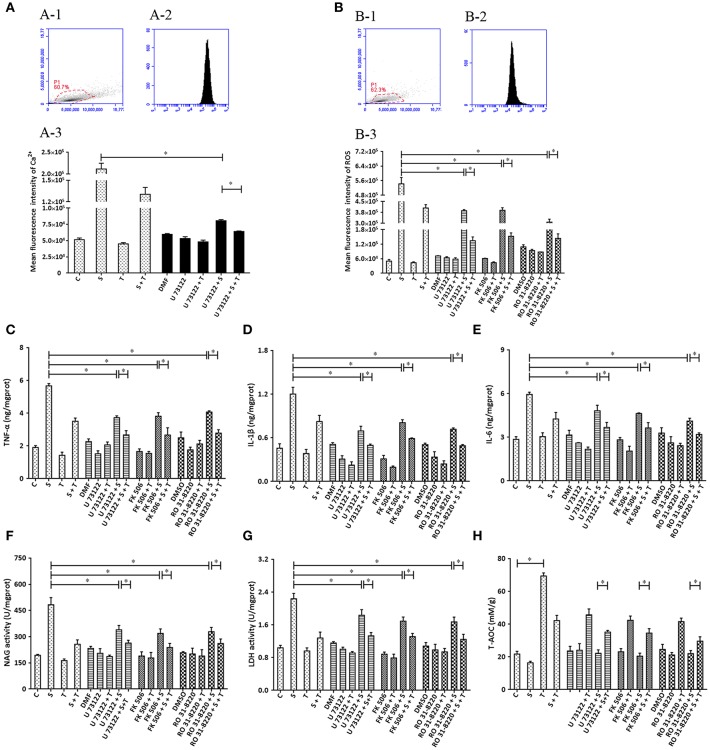
The influence of the inhibitors of PLC (U73122), CaN (FK506), and PKC (RO31-8220) on the effect of taurine on inflammatory responses induced by *S. uberis* in MAC-T cells. MAC-T cells were exposed to pretreatment inhibitors (U73122, FK506, or RO31-8220) for 1 h, and then administrated taurine for 24 h. Subsequently, cells were infected with *S. uberis* in mid-exponential phase at a multiplicity of infection (MOI) of 10 for 3 h at 37°C. Intracellular Ca^2+^ concentration was evaluated by staining cells with Fluo-3 AM. 10,000 cells per sample were analyzed using Cell Quest Pro acquisition and analysis software. Gating of the cell population (A-1), pictorial diagram (A-2), and statistic results (A-3). **(B–H)** Cells were infected with *S. uberis* in mid-exponential phase at a multiplicity of infection (MOI) of 10 for 4 h at 37°C. **(B)** Intracellular ROS content was evaluated by staining cells with DCFH-DA. 10,000 cells per sample were analyzed using CellQuest Pro acquisition and analysis software. Gating of the cell population (B-1), pictorial diagram (B-2), and statistic results (B-3). The production of cytokines in the supernatant was measured by ELISA kits. **(C)**: TNF-α; **(D)**: IL-1β and **(E)**: IL-6. NAGase and LDH in supernatant were determined using commercial kits. **(F)**: NAGase activity and **(G)**: LDH activity. **(H)** The cells were used to detect antioxidant capacity (T-AOC) by a commercial kit. Statistic results are representative of 3 independent experiments. Data are presented as the means ± SEM. ^*^(*P* < 0.05) = significantly different between the indicated groups.

Based on the ability of siRNA to disturb the expression of target genes (PLC, CaN and PKC), various inhibitors (U 73122, FK506 and RO 31-8220) were applied and biochemical parameters related to inflammation assayed. The results indicate that U 73122 down-regulates intracellular Ca^2+^ elevated by *S. uberis* challenge. A significant difference was observed between the *S. uberis* challenge group and the U 73122 + *S. uberis* group (*P* < 0.05). Taurine reduced Ca^2+^ levels both in U 73122 pretreated and untreated *S. uberis* infected groups (*P* < 0.05) (Figure 8A). The patterns of these 3 inhibitors to inflammation and cell injury are similar to those siRNAs targeting PLC, CaN and PKC. In summary, blocking PLC, CaN, and PKC with inhibitors decreased intracellular ROS; TNF-α, IL-1β, IL-6; NAGase, and LDH in cell culture supernatants. Taurine suppressed these indexes whether the inhibitors were present or not (Figures 8B–H).

## Discussion

**Figure 9:**
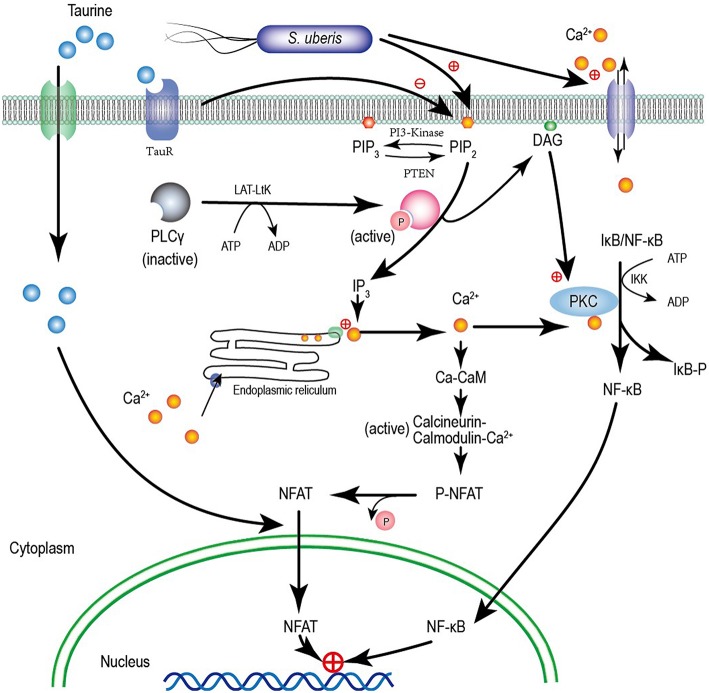
A model for taurine regulating the *S. uberis* induced inflammatory responses via the phosphoinositides/Ca^2+^ systems which followed by the Ca^2+^-PKCα-NF-κB, and Ca^2+^-CaM-CaN-NFAT signaling pathways.

Calcium has long been considered the most common message carrier in cells (38–40). The data presented here are consistent with our previous study demonstrating significantly elevated cytoplasmic Ca^2+^ by *S. uberis* infection (14). This is associated with the activation of PLC and hydrolyzation PIP2 to produce IP3 which bonds to Ca^2+^ channels allowing Ca^2+^ entry (41, 42). We found that the expression and activation of PLCγ1 the main isoform of PLC in epithelial cells (43) and cytoplasmic IP3 increases after *S. uberis* challenge. NCX1 which allows bidirectional Ca^2+^ crossing of cell membranes is unaltered. Taurine protects against various infection induced injuries and may be linked to the regulatory role of taurine in cytoplasmic Ca^2+^ levels. Taurine significantly decreases intracellular Ca^2+^, but its competitive inhibitor β-alanine has no effect on the down-regulation of taurine. Although special siRNA targeting to TauT and PAT1 have been used, taurine still significantly decreases intracellular Ca^2+^ concentration after *S. uberis* challenge These data indicate that taurine does not need to enter cytoplasm to regulate intracellular Ca^2+^. A model for taurine regulating the *S. uberis*-induced inflammatory responses via the phosphoinositides/Ca^2+^ systems was showed in Figure 9.

PIs are a minority family of acidic phospholipids in cell membranes. They are known as lipid regulators of membrane proteins. The signature PI attracts a specific complement of functionally important, loosely attached, peripheral membrane proteins that cooperate with other signal recognition proteins (44). PIP2 and PIP3 are 2 principal phosphoinositides. Regulation of their levels at the plasma membrane plays a critical role in the pathogenic mechanism of some bacterial pathogens. *Enteropathogenic Escherichia coli* (EPEC) infection subverts PIP2 and PIP3 and modulates cell death and innate immunity (45). *S. uberis* challenge significantly increases PIP3 levels and decreases PIP2. PLC, which breaks down PIP2 to IP3 and phosphatidic acid, and by phosphorylation of the inositol ring in the 3′ position by PI3K, generates PIP3 in the infection site. Our data confirms detection of PLC and IP3. The expression and phosphorylation of PI3K and PTEN, which are key enzymes that mediate the switch of PIP2 and PIP3 are elevated after *S. uberis* infection. This suggests complete metabolism of phosphoinositides by MAC-T cells. Pretreatment with taurine increases PIP2 and decreases PIP3 with *S. uberis* challenge. PI3K and PTEN levels are also moderated by taurine. We assume that taurine transfers the extracellular nutrient signal to trigger intercellular innate immunity.

NF-κB is an important transcription factor regulating expression of many genes. Upon activation by various factors, NF-κB translocates to the nucleus (46). Our observations suggest that NF-κB activity is up-regulated as a result of *S. uberis* infection. Employing immunofluorescence analysis, we detected increased NF-κB in nuclear regions. Utilizing DNA-Ag nanocluster molecular beacons and exonuclease III-assisted signals, the DNA binding signal was increased for only activated NF-κB. The phosphorylation levels of IKKα and IκBα were elevated in MAC-T cells. Mean IκBα was activated by IKKα; IκBα detaches from the NF-κB dimer which is then free to enter the nucleus. Protein kinase C (PKC, EC 2.7.11.13), is a family of protein kinase enzymes. It is activated by Ca^2+^ signaling and regulates metabolism, cell division, apoptosis and other cell functions. Recently, it has been shown that PKC is closely linked to the inflammatory response through Ca^2+^ via the influence of NF-κB. PKC-α is unique within the PKC family because its primary mode of regulation involves its interaction with the cell membrane. It plays a vital role in epithelial tissue (47, 48). Taurine negatively affects the concentration and activity of PKCα in *S. uberis* challenged bovine MECs. It seems that the PLCγ1-IP3- Ca^2+^-PKCα-NF-κB signaling pathway is involved the bioprocesses of *S. uberis* infection and the regulatory role of taurine.

In contrast with NF-κB, NFAT is a multiply faceted transcription factor involved in inflammatory regulation. The precise roles of NFAT are pathogen and cell type dependent. Zanoni et al. found that CD14 signaling triggers the CaN/NFAT pathway in DCs but not in macrophages strongly indicating that NFAT has distinct roles in different cells (49). Our previous study reported that in mouse MECs, *S. uberis* provokes NFAT activation and taurine alleviates this process. This process is connected with alteration of cytoplasmic Ca^2+^. This phenomenon is validated in the current study in MAC-T cells. CaN and GSK-3, which are known to coordinate the import and export of NFAT, were detected. They were markedly activated by *S. uberis;* taurine subverts these changes. CaN and GSK-3 take part in inflammatory regulation. The enhancing role of GSK-3 kinases in CaN signaling has been reported in *Staphylococcus aureus* and other inflammatory diseases. CaM, a bridge protein between Ca^2+^ signaling and Ca^2+^ regulating proteins/enzymes, had a similar change. Taken together, the PLCγ1-IP3- Ca^2+^-CaM-CaN-NFAT signaling pathway may mediate taurine regulation of *S. uberis* infection.

Transcription factors NF-κB and NFAT initiate the inflammatory cascade characterized by the overexpression ROS, RNS, cytokines and other inflammatory mediators. The induced cell damage accompanies the elimination of pathogens if uncontrolled. Our results suggest that intracellular ROS, iNOS, and NO are significantly increased after *S. uberis* challenge. Taurine pretreatment significantly downregulates their production in MAC-T cells. Forty cytokines/molecules in cell culture supernatants were simultaneously assayed by protein microarray. *S. uberis* elevated the expression of most pro-inflammatory cytokines (10/13), anti-inflammatory cytokines (4/5) and taurine largely inhibited these changes. TNF-α, IL-1β, and IL-6, 3 important pro-inflammatory cytokines, are widely accepted as downstream factors subsequent to pathogen infection and demonstrated a similar pattern. These data indicate that the inflammatory responses were fully activated following *S. uberis* targeting and excitation of PIs/Ca^2+^ systems. Taurine alleviated these bioprocess as confirmed by the detection of the cell injury biomarkers NAGase and LDH.

The results and phenomena described above denote an integration of all involved active signaling pathways. To ensure that phosphoinositids/Ca^2+^ systems play an important role in taurine regulation of the inflammatory response associated with *S. uberis* infection and the importance of PLCγ1-IP3-Ca^2+^-PKCα-NF-κB, and PLCγ1-IP3- Ca^2+^-CaM-CaN-NFAT signaling pathways, siPLC, siCaN, and siPKC were used to inhibit the two signaling pathways through interference with the expression of the target proteins. SiPLC decreased intracellular Ca^2+^ resulting from *S. uberis* challenge. Taurine performed this function whether or not siPLC was present. Inhibitory rates were lower when siPLC was present. ROS, cytokines, NAGase, and LDH had similar patterns. There were no significant differences in the regulatory roles of taurine in siPLC, siCaN, and siPKC systems. The phosphoinositides/Ca^2+^ systems participated in taurine regulating *S. uberis*-induced inflammation. Both Ca^2+^-PKCα-NF-κB, and Ca^2+^-CaM-CaN-NFAT mediated this process. It was further confirmed by the use of three inhibitors of U73122, FK506, and RO31-8220 which restrained the activity or function of PLC, CaN, and PKC, respectively.

In summary, *S. uberis* induced inflammatory responses are elicited through lipid products mainly by IP3 that acts on membrane phosphoinositides and activate the Ca^2+^-PKCα-NF-κB, and Ca^2+^-CaM-CaN-NFAT signaling pathways. There is negative regulation of these inflammatory responses by taurine via the phosphoinositides/Ca^2+^ systems. These data augment our understanding of the mechanisms of multifunctional nutrient taurine attenuation of the inflammatory responses causing by *S. uberis* infection, and provides theoretical support for the prevention of this disease.

## Data Availability

Requests to access the datasets should be directed to Miao Jinfeng, miaojinfeng@njau.edu.cn.

## Author Contributions

ML and PX performed the whole experiments and wrote the manuscript. YX and XH participated in the design of this study. YX and ZW provided assistance for data acquisition, data analysis, and statistical analysis. WR collected important background information. VP performed manuscript review. JM carried out the definition of intellectual content and provided the support platform and funding. All authors read and approved the final manuscript.

### Conflict of Interest Statement

The authors declare that the research was conducted in the absence of any commercial or financial relationships that could be construed as a potential conflict of interest.
